# Mechanical properties and energy evolution characteristics of fissure sandstone under the interaction between water and fissures

**DOI:** 10.1371/journal.pone.0351174

**Published:** 2026-06-09

**Authors:** Qingqing He, Jianhua Deng, Guixin Yuan

**Affiliations:** School of Civil Engineering, Guizhou University, Guiyang, Guizhou Province, China; Henan Polytechnic University, CHINA

## Abstract

To investigate the mechanical properties and damage evolution of fissure sandstone under the interaction between water and fissures, this study performed uniaxial compression tests on sandstone specimens with different water conditions (dry, natural, and saturated) and fissure angles (0°, 30°, 45°, 60°, and 90°). The experimental results indicate that peak strength decreased markedly with increasing water content, with reductions of 28.68%–53.99% under saturated conditions relative to dry conditions. In contrast, peak strength increased progressively with fissure angle. Crack initiation stress and crack damage stress exhibited similar trends. Based on the normalized ratios of characteristic stress, two damage evaluation indices, $R_{ci}$ and $R_{cd}\;$, were proposed to characterize the weakening effect of fissures on rock bearing capacity during the crack initiation and crack propagation stages, respectively. The energy evolution results show that the strain energy corresponding to characteristic stress decreases significantly with increasing water content and generally increases with fissure angle. In addition, this study introduced a warning coefficient λ based on the ratio of elastic strain energy to dissipated strain energy to identify precursor information associated with rock failure. The results show that λ increased with water content and varied with fissure angle in an M-shaped pattern, with significant peaks at 30° and 60°. Under saturated conditions, water exerted the strongest effect on mechanical parameters at a fissure angle of 0°, while the overall effect remained relatively small at 30°. These findings provide a valuable reference for risk assessment and disaster prevention in geotechnical engineering.

## 1. Introduction

In natural geological environments, rock masses usually contain various structural planes, such as joints, fissures, and bedding planes. Among these structural features, fissures play a critical role in controlling the strength and deformation characteristics of rock masses and also act as preferential channels for water seepage [1,2]. As water infiltrates into the interior of the rock mass along fissures, the interaction between water and fissures intensifies crack propagation and connectivity, which further weakens the mechanical properties of rocks and may induce geological disasters and engineering accidents, including landslides, dam foundation deformation, and mine water inrush. Accordingly, the coupling effect between water and fissures has attracted extensive attention in engineering geology and disaster prevention research [3].

The influence of water content on the mechanical properties of rocks was widely investigated, and numerous studies were conducted in this field. It was demonstrated that water significantly affected the mechanical properties of rocks through physicochemical mechanisms, which were primarily manifested in the degradation of peak strength and elastic modulus, together with the intensification of plastic deformation and initial damage [4]. Furthermore, the movement and accumulation of water within rocks redistributed the stress field [5,6]. Water was found to negatively affect the mechanical properties of rocks [7,8]. Zhang et al. [9] observed that the peak strength of rocks initially increased and subsequently decreased with increasing water content, with the corresponding critical water contents varying among samples. Tang et al. [10] examined the decay behavior of the uniaxial compressive strength of mudstone under varying water contents and demonstrated that the uniaxial compressive strength decreased exponentially as the water content increased. Through uniaxial cyclic compression tests on saturated and dry red sandstone specimens, Zhang and Gao [11] revealed that the energy accumulation limit of saturated sandstone decreased by 38.9%, whereas both its dissipated energy and residual elastic energy increased, which consequently reduced the amplitude and rate of energy release. Cai et al. [12] performed a comparative analysis on the water-induced softening mechanisms of granite, sandstone, and marble. It was found that the failure modes of sandstone and granite transformed from a tensile-dominated mode under dry conditions to a shear-dominated mode under saturated conditions, which was attributed to the weakening of intergranular friction caused by water. In contrast, the failure mode of marble remained largely unchanged due to its compact crystalline texture. Yu et al. [13] combined laboratory experiments with a three-dimensional grain-based model (GBM) to systematically analyze the influence of initial water content on the mechanical properties and failure behavior of rocks. The results demonstrated that both crack initiation stress and crack damage stress exhibited a linear decreasing trend with increasing water content. Luo et al. [14] carried out uniaxial compression tests on sandstone under different water conditions, focusing on the energy storage characteristics and rock burst susceptibility of the specimens. This work provided new insights into how water reduces rock burst susceptibility.

With the advancement of geotechnical engineering into deep underground regions, the environmental conditions of engineering rock masses were becoming increasingly complex [15]. Although the aforementioned studies provided valuable insights into rock mechanical behavior, they were mainly conducted on intact rock specimens and did not fully consider the widespread defects in natural rock masses. In contrast, natural rock masses generally contain multiple defects such as joints, fissures, and bedding planes, resulting in pronounced anisotropy in strength, deformation, crack evolution, and failure patterns. In this study, artificially created fractures are called ‘fissures’, whereas the cracks produced by mechanical loading are called ‘cracks’ to avoid confusion. The stress redistribution induced by underground excavation was prone to aggravate the development of these pre-existing defects, thereby initiating, propagating, and coalescing fissures and ultimately causing deformation and instability of the surrounding rock walls [16,17]. Huang et al. [18] and Li et al. [19] compared the mechanical properties of intact and pre-fissured specimens under uniaxial compression and observed that both the uniaxial compressive strength and elastic modulus of the pre-fissured specimens were lower than those of the intact ones. Zhao et al. [20] systematically analyzed the mechanical behavior and failure patterns of specimens containing prefabricated fissures through uniaxial compression tests and revealed that the pre-fissured specimens exhibited more evident progressive failure characteristics than the intact ones. Wong et al. [21] and Bobet et al. [22] observed through experiments that tensile cracks first initiated at the crack tips under uniaxial compression, followed by the development of secondary cracks. Li et al. [23] classified the fissure propagation process into five evolutionary stages—compaction, linear elasticity, stable crack propagation, accelerated crack propagation, and post-peak residual—and defined the stresses corresponding to the transition points between these stages as characteristic stresses. Chen et al. [24] utilized the particle flow code (PFC 2D) to construct numerical models of sandstone with varying fissure angles and strain rates, and systematically analyzed the characteristic stress, crack evolution processes, and energy damage characteristics under different conditions.

In fact, pre-existing fissures within rock masses not only reduce the mechanical properties of rocks to a considerable extent but also create preferential channels for water seepage. As water infiltrates through these fissures, the interaction between water and fissures further accelerates the deterioration process of rock masses, which may ultimately compromise the stability of engineering rock masses. To thoroughly explore the mechanical behavior and failure mechanism of fissure rock masses under varying water contents, Yu et al. [25] performed uniaxial compression tests and systematically analyzed the mechanical properties and crack propagation behavior of siltstone specimens with different fissure angles and water contents. Yang et al. [26] found that the peak strength and elastic modulus of water-saturated fissure sandstone were considerably lower than those of the dry specimens. Wang et al. [27] examined the effects of water content and inclination angle on the shear behavior of sandstone, revealing that the inclination angle had the most pronounced impact on the shear failure mode, whereas the influence of water content was relatively minor. Through uniaxial compression tests, Song et al. [28] investigated the progressive failure behavior of fissure sandstone and observed a significant reduction in both strength and elastic modulus under saturated conditions. Hu et al. [29] examined the mechanical properties of fissure limestone under long-term water–rock coupling and elucidated the mechanisms of energy dissipation and damage evolution within the water environment. Zhang et al. [30] demonstrated that the deterioration effects at fissure tips induced by water or chemical solutions were considerably more pronounced than those caused by air erosion. Zang et al. [31] conducted uniaxial compression, Brazilian splitting, and direct shear tests to investigate the mechanical response and failure modes of specimens with prefabricated fissures under the coupling effect of water content and lithology. The study revealed the failure mechanisms associated with water–rock interaction and clarified the internal mechanism governing strength degradation and damage mitigation. Shi et al. [32] performed a comparative analysis of the mechanical properties, energy dissipation behavior, and failure modes of prefabricated fissure sandstone under different water conditions. The results indicated that the damage modes changed systematically with variations in water conditions. Zhang et al.[33] conducted graded cyclic loading–unloading tests on rocks with different water contents and crack numbers, and evaluated the sensitivity of the bearing behavior of fissure rocks to water content. The findings showed that as water content increased, both peak strength and crack initiation stress decreased, accompanied by reductions in elastic strain energy and dissipated strain energy at the peak stress state.

Although numerous studies have investigated the effects of water conditions and fissure angles on rock behavior, the damage and instability mechanisms of rocks under the interaction between water and fissures have not been fully clarified. Most existing studies focus on a single influencing factor, either analyzing the effects of water conditions on the mechanical properties of rocks with a fixed fissure angle or examining the role of fissure angle in controlling crack propagation and failure modes. Consequently, the combined influence of water conditions and fissure angles on rock mechanical properties still requires further investigation. Fissures not only act as seepage pathways for water migration but also serve as important regions for stress concentration and crack initiation. Furthermore, the softening and lubrication effects of water can significantly alter the stress distribution at fissure tips, crack propagation behavior, and energy evolution characteristics. Therefore, water conditions and fissure geometry jointly govern the mechanical response and instability behavior of fissure rock masses. Previous studies have mainly concentrated on peak strength, elastic modulus, and macroscopic failure patterns, whereas limited attention has been paid to crack initiation stress, crack damage stress, and energy evolution characteristics. Accordingly, this study performed uniaxial compression tests on sandstone specimens with different fissure angles under saturated, natural, and dry conditions. The study investigated the characteristic stress, energy storage characteristics, precursor characteristics before critical failure, and failure behavior of the specimens. Particular attention was given to: (1) the influence of the interaction between water and fissures on peak strength, crack initiation stress, crack damage stress, and failure modes; (2) the evolution of energy accumulation and dissipation under varying water conditions and fissure angles. The findings provide valuable references for the stability evaluation and disaster prevention of fissure rock mass engineering in water-rich environments.

## 2. Experiments and methodology

### 2.1. Specimen preparation

**Fig 1:**
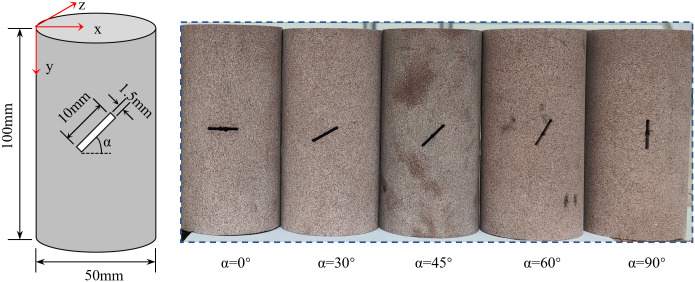
Sandstone specimens containing a pre-existing single fissure.

The sandstone specimens for this study were obtained from Jining, Shandong Province, and were prepared from a single intact block. The sandstone exhibited initial physical properties of 14.31% porosity, 2.34 g/cm³ oven-dried density, 2.43 g/cm³ water saturated density, and a P-wave velocity of 2.78 km/s. In accordance with ISRM standards [34], the rock mass was cored, cut, and polished into cylindrical specimens with a diameter of 50 mm and a height of 100 mm. The two end faces were parallel within 0.01 mm and perpendicular to the specimen axis. To reduce specimen variability, we selected sandstone samples with similar wave velocities and sound appearance. Six specimens were randomly assigned as intact controls, and the remaining samples were used to prepare fissure specimens using a high-pressure water jet cutting method. Specifically, a 1.5 mm diameter hole was drilled at the center of each specimen using a high-pressure water jet. A mechanical cutter was then inserted into the hole to create a fissure with a length of 10 mm and a width of 1.5 mm [33,35]. Fissures were prepared at five angles relative to the horizontal axis: 0°, 30°, 45°, 60°, and 90°. Six specimens were prepared for each angle. Fig 1 presents the details of the fissure sandstone specimens.

**Fig 2:**
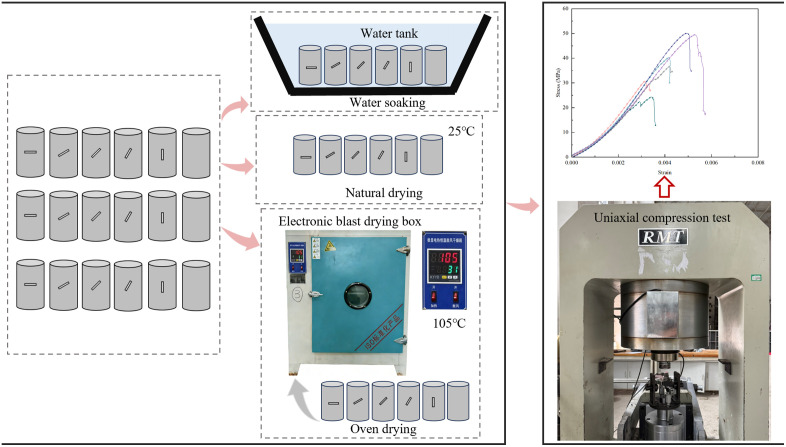
Schematic diagram of the processing steps for fissure rock specimens.

As shown in Fig 2, Upon completion of specimen fabrication, all specimens were divided into three groups and conditioned to achieve oven-dried states, natural states and water saturated states as follows: (1) the first group was placed in a constant-temperature oven at 105°C for 48 hours, resulting in oven-dried sandstone specimens; (2) the second group was maintained at room temperature (25°C) before testing and was designated as natural sandstone specimens; (3) the third group was fully immersed in water for 48 hours to produce water saturated sandstone specimens. For each test condition, we tested two parallel specimens, and we used their average values as the final results.

### 2.2. Experimental system

In this study, uniaxial compression tests were performed on the RMT-301b Rock and Concrete Mechanics Testing System, which integrated data acquisition and stress-loading functions, supported a maximum hydraulic load of 1500 kN, and possessed a high-rigidity structure to reduce systematic errors. The testing system provided both stress control and displacement control. We adopted displacement – controlled loading with a loading rate of 0.05 mm/min and used a loading head with a precision of 0.0001. During the tests, we measured the axial strain and radial strain of the specimens using a vertical pressure sensor and a horizontal displacement sensor, with data recorded every 2 seconds. Before assembling the specimens, we applied a uniform layer of lubricant to both ends of the specimens to reduce the effect of end friction.

## 3. Results analysis

### 3.1. Stress–strain curve

**Fig 3:**
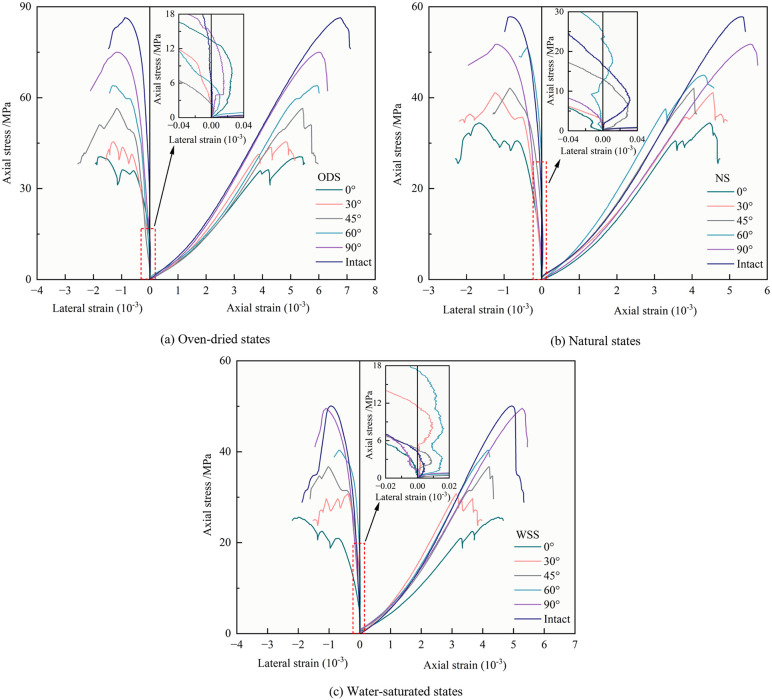
Stress–strain curves of fissure rock specimens.

Fig 3 illustrates the stress–strain responses of rock specimens with different fissure angles under oven-dried states, natural states, and water-saturated states. It was observed that, under identical water content, specimens with varying fissure angles exhibited comparable trends in their stress–strain evolution. Throughout loading, all specimens passed through the compaction, elastic, plastic, and post-peak stages. During compaction, pore closure under applied load produced a characteristic concave curve, reflected by a progressively increasing slope. In the elastic stage, the slope of the stress–strain curves remained nearly constant, demonstrating linear elastic behavior in agreement with Hooke’s law. In the plastic yielding stage, the curves exhibited nonlinear behavior, the slope decreased, and the stress increment approached a plateau.

### 3.2. Strength and deformation characteristics

**Fig 4:**
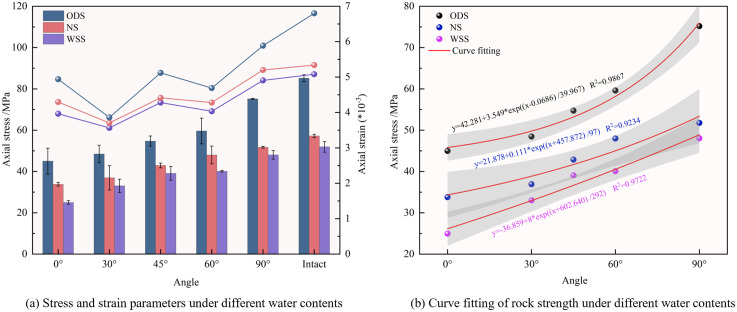
Effects of different water contents on the stress and strain parameters.

The peak strength and peak strain were identified to quantitatively assess the strength and deformation characteristics of fissure sandstone under three water content states. As shown in Fig 4(a), under the same water conditions, the peak strength gradually increased as the fissure angle increased. Fig 4(b) illustrates a nonlinear fitting relationship between peak strength and fissure angle, indicating that this nonlinear relationship became more pronounced as the water content decreased. This behavior could be attributed to the fact that, under saturated conditions, hydration reduces friction between rock particles and decreases contact stress along fissure surfaces. As a result, the sensitivity of peak strength to fissure angle weakens, leading to a more linear increase of peak strength with fissure angle. In contrast, under dry conditions, higher friction and interlocking forces exist along fissure surfaces, and the geometric effect of fissure structures becomes dominant. Consequently, the nonlinear relationship between peak strength and fissure angle becomes more significant as the fissure angle increases. The peak strength decreased from 44.98–75.17 MPa in the oven-dried state to 24.97–51.95 MPa in the water-saturated states, with reductions of 28.68%–53.99% under saturated states relative to dry states. Relative to the natural states, oven-dried states substantially enhanced the load-bearing capacity of sandstone specimens with varying fissure angles. These findings indicated that water content significantly affected the peak strength, following the sequence ODS > NS > WSS, and that water saturation substantially weakened the load-bearing capacity of fissure sandstone.

The peak strain decreased from 0.00386–0.00589 in the oven-dried state to 0.00357–0.00491 in the water-saturated state, with reductions of 7.64%–19.74% under saturated states relative to dry states, which indicated that increasing water content substantially diminished the rock’s ability to resist deformation. The peak strain exhibited a distinct “W”-shaped evolution trend with increasing fissure angle under oven-dried, natural, and water-saturated states. At a fissure angle of 0°, the axial stress mainly acted perpendicular to the fissure surface, generating strong normal compression on the fissure plane. Under this condition, crack propagation required overcoming greater resistance, resulting in relatively high peak strain. As the fissure angle increased to 30°, the shear stress component acting on the fissure surface gradually increased, while the tensile stress concentration region shifted toward the fissure tips. This stress redistribution accelerated crack propagation and reduced the deformation before failure, leading to lower peak strain. At a fissure angle of 45°, the fissure tips experienced coupled tensile–shear stresses, which produced more complex crack propagation paths and improved the coordinated deformation capability of the rock, thereby slightly increasing the peak strain. When the fissure angle further increased to 60°, the shear stress component became stronger and promoted sliding along the fissure surface, causing peak strain to decrease again. At a fissure angle of 90°, the fissure direction became nearly parallel to the loading direction, and compressive stress maintained the fissure surfaces in a closed state. Consequently, the crack propagation paths were constrained, resulting in relatively high peak strain. Furthermore, under water saturated states, pore water pressure and the lubrication effect of water molecules further decreased the effective normal stress on fissure surfaces and enhanced fissure sliding capacity. These effects facilitated crack initiation and propagation, causing the peak strain under water-saturated states to remain lower than that under oven-dried states.

The fissure-induced damage rate of fissured rock specimens was defined as the reduction in peak strength relative to intact specimens. As shown in Table 1, the maximum damage rate occurred at a fissure angle of 0°, reaching 52.42%, 40.92%, and 51.94% under oven-dried natural and water-saturated states, respectively, whereas the minimum damage rate was observed at a fissure angle of 90°, with values of 11.76%, 15.37%, and 7.51%. The damage rate decreased progressively with increasing fissure angle across all water content states. Moreover, the fissure-induced damage in the oven-dried state was consistently higher than in the water-saturated states, indicating that although the oven-dried specimens had higher peak strength, fissures inflicted more pronounced damage on sandstone under oven-dried states.

**Table 1:** Damage rates of peak strength for rock specimens under varying water states and fissure angles.

Water condition	Fissure angle	Damage rate
Oven-dried states	0°	52.42%
	30°	43.12%
	45°	35.77%
	60°	30.03%
	90°	11.76%
	Intact	–
Natural states	0°	40.92%
	30°	35.46%
	45°	26.47%
	60°	16.11%
	90°	15.37%
	Intact	–
Water-saturated states	0°	51.94%
	30°	36.41%
	45°	29.35%
	60°	22.87%
	90°	7.51%
	Intact	–

The findings demonstrated that the fissure angle was a significant factor controlling the peak strength of rock specimens. As the fissure angle increased, specimens under varying water content conditions displayed comparable damage evolution characteristics during uniaxial compression. When the fissure angle was less than 30°, the damage rates of specimens in oven-dried states, natural states and water saturated states exceeded 30%. In geotechnical engineering contexts, stresses from mining, construction, dynamic loads from blasting, and substandard operations could collectively compromise rock integrity and promote fissure development. Regions where fissures formed at substantial deviations from the stress loading direction (α ≤ 30°) necessitated prioritized reinforcement and intensified monitoring strategies.

### 3.3. The characteristics of characteristic stress

Characteristic stress was defined as the stress at the critical points of each stage in the progressive failure of fissure sandstone, and this section emphasized the evaluation of crack initiation stress and crack damage stress. During most of the experiments, positive lateral strain emerged under low axial stress due to micro-porosity compression, as presented in Fig 3. Accordingly, the MLSR method [36] was employed to identify the crack initiation stress, and the volumetric strain method [37] was utilized to determine the crack damage stress.

**Fig 5:**
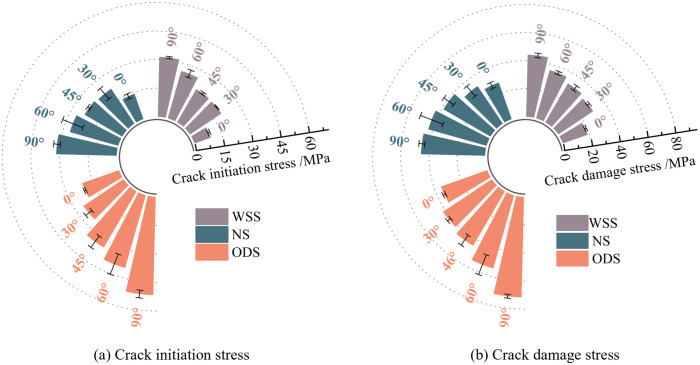
Characteristic stresses of fissure sandstone specimens under different saturation states.

Based on the described methods, the crack initiation stress and crack damage stress were identified, as presented in Fig 5. The crack initiation stress decreased from 20.53–58.17 MPa under oven-dried states to 9.09–33.31 MPa under water saturated states, corresponding to a decrease of 21.78%–55.73%. The crack damage stress decreased from 34.94–80.15 MPa to 18.83–49.32 MPa, with a decrease range of 28.68%–46.11%. The findings demonstrated that, for the same fissure angle, both crack initiation stress and crack damage stress decreased with increasing water content, whereas for the same water content, both stresses increased with the fissure angle. This phenomenon was attributed to the penetration of water molecules into internal pores and fissures, which reduced the cohesion between mineral grains and softened the cementing materials, leading to a deterioration in the mechanical properties of the sandstone.

Based on the results of uniaxial compression tests on intact sandstone specimens, we obtained the mechanical property parameters (crack initiation stress and crack damage stress), as summarized in Table 2. Under different water conditions, both parameters exhibited relatively small standard deviations, indicating good homogeneity of the intact sandstone specimens. The differences among the intact sandstone specimens mainly arose from variations in water content. As water content increased, both crack initiation stress and crack damage stress gradually decreased, demonstrating that the presence of water significantly weakens the mechanical properties of the rock material.

**Table 2:** Mechanical test results of intact sandstone specimens under different water content conditions.

Condition	Specimen No.	$\sigma_{cI}$ (MPa)	${\overset{―}{\sigma }}_{cI}\pm SD$ (MPa)	$\sigma_{cD}$ (MPa)	${\overset{―}{\sigma }}_{cd}\pm SD$ (MPa)
Oven-dried states	D-1	53.72	58.17 ± 6.30	76.26	80.16 ± 5.50
	D-2	62.63		84.03	
Natural states	N-1	39.79	39.36 ± 0.64	55.55	55.39 ± 0.24
	N-2	38.89		55.22	
Water-saturated states	S-1	31.44	33.31 ± 2.64	47.75	49.32 ± 2.23
	S-2	35.17		50.89	

Note: “SD” represents “standard deviation.”

In accordance with damage mechanics theory, fissures in the rock mass caused stress concentrations and redistribution, leading to a decline in the macroscopic mechanical properties of the rock. This study defined the initial stress reduction parameter $R_{ci}$ and the damage stress reduction parameter $R_{cd}$ to assess the extent to which fissures weakened the rock’s characteristic stresses. The approaches for determining $R_{ci}$ and $R_{cd}$ were as follows:


$$\begin{array}{c} R_{ci}=\frac{\sigma_{\text{cI}}-\sigma_{\text{c}i}}{\sigma_{\text{cI}}} \end{array}$$ (1)

$$\begin{array}{c} R_{\text{cd}}=\frac{\sigma_{\text{cD}}-\sigma_{\text{cd}}}{\sigma_{\text{cD}}} \end{array}$$ (2)

In this context, $\sigma_{ci}$ and $\sigma_{cd}$ were the crack initiation stress and crack damage stress of the fissure specimens, while $\sigma_{cI}$ and $\sigma_{cD}$ were those of the intact specimens.

In rock damage mechanics, damage variables are commonly established based on elastic modulus degradation, strength deterioration, and strain evolution to characterize the damage evolution process from the initial state to failure. The damage variable $D_{E}\;$, defined using elastic modulus reduction ($D_{E}=\text{1}-E_{d}/E_{\text{0}}$, where $E_{d}$ denotes the elastic modulus of fissured rock specimens and $E_{\text{0}}$ denotes that of intact rock), reflects the degradation effect of fissures on rock stiffness during the elastic stage. However, because this parameter only depends on the linear elastic portion of the stress–strain curve, it cannot describe crack initiation or crack propagation behavior. By comparison, the $R_{ci}$ and $R_{cd}$ are stage-related weakening parameters derived from two key characteristic stress points, namely crack initiation stress and crack damage stress. These parameters independently characterize the stress response during the crack initiation stage and the crack propagation stage, respectively, and can therefore capture the weakening effects at crack evolution stages that $D_{E}\;$ cannot reflect. Specifically, $R_{ci}$ represents the weakening effect of fissures on the bearing capacity during crack initiation, while $R_{cd}$ characterizes the influence of fissures during stable crack propagation. Their physical significance is to quantify the influence of fissures on characteristic stress. Since $D_{E}$ describes deterioration during the elastic stage, whereas $R_{ci}$ and $R_{cd}$ characterize deterioration during the nonlinear crack evolution stage, these parameters exhibit strong complementarity.

In addition, $R_{ci}$ and $R_{cd}$ only require two clearly identifiable characteristic stress points ($\sigma_{ci}$ and $\sigma_{cd}$) from the stress-strain curve, resulting in a simple and robust parameter determination process. Therefore, $R_{ci}$ and $R_{cd}$ are suitable for evaluating the influence of fissures on crack initiation stress and crack damage stress. Furthermore, the normalization between fissured and intact specimens more intuitively reveals the influence of fissure angle on rock mechanical properties. In this study, larger values of $R_{ci}$ and $R_{cd}$ indicate stronger weakening effects of fissures on crack initiation stress and crack damage stress, implying more severe deterioration and a more unstable mechanical state.

**Fig 6:**
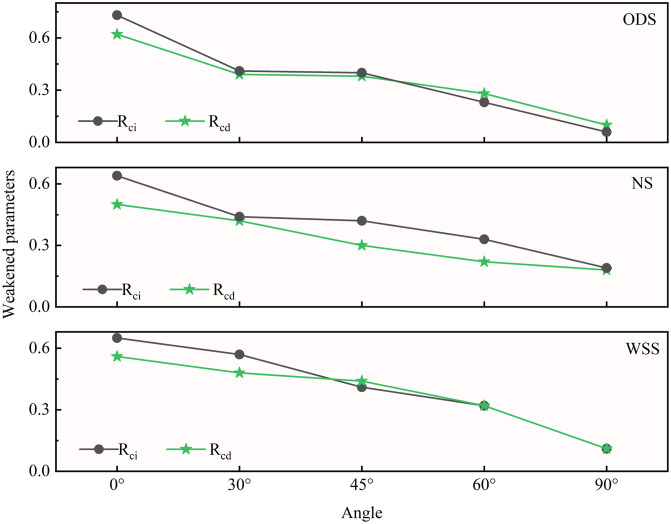
Characteristic stress reduction parameters of fissure rock specimens in various water states.

As illustrated in Fig 6, the characteristic stress weakening parameters of sandstone specimens were examined under varying fissure angles and water content conditions. The findings revealed a negative correlation between fissure angle and the extent of characteristic stress weakening across different water states. Notably, the fissures exerted a more pronounced weakening effect on crack initiation stress than on crack damage stress in all three water conditions. This was mainly attributed to the early loading stage, during which the applied stress was insufficient to induce rock deformation, resulting in significant stress concentration near fissure tips and early microcrack activation, thereby exerting a greater impact on the crack initiation phase.

### 3.4. Energy evolution

#### 3.4.1. Pre-peak stress energy evolution characteristics.

**Fig 7:**
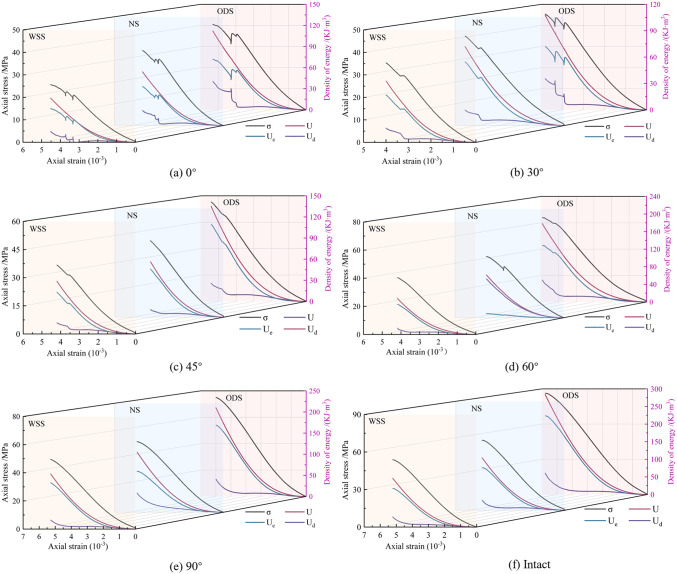
Energy evolution characteristics of fissure rock specimens under different water conditions.

Based on the strain energy parameter calculation method [38], the energy evolution curves of sandstone under the coupling effects of water content and fissure angle were obtained, as shown in Fig 7. After treatments with different water conditions, the total strain energy U of fissure sandstone continuously accumulated with the increase of axial strain. The evolution of elastic strain energy $U_{e}$ was strongly and positively correlated with the corresponding stress–strain curve, while the dissipated strain energy $U_{d}$ underwent three distinct stages of evolution: a slow increase, a relatively stable stage, and a rapid rise before the peak stress.

At the initial loading stage, the total strain energy U absorbed by the rock specimen was mainly converted into dissipated strain energy $U_{d}$ due to the closure of pre-existing microcracks and pores within the rock. During the elastic deformation stage, the total strain energy U was predominantly accumulated in the form of elastic strain energy $U_{e}$ inside the rock, while the proportion of dissipated energy remained nearly constant, indicating that few new cracks were generated and the energy loss ratio was low. As the axial strain continuously increased, the stress–strain curve exhibited a nonlinear rising trend, and the specimen entered the yielding stage. The number and propagation of internal microcracks gradually increased, leading to irreversible plastic deformation, and dissipated strain energy $U_{d}$ significantly increased during this stage. In the unstable failure stage, with the continuous increase of external loading, the microcracks within the specimen progressively propagated and interconnected, leading to the formation of a macroscopic fracture surface on the specimen. The elastic strain energy $U_{e}$ stored in the specimen was rapidly released and converted into the dissipated energy driving crack propagation, thereby inducing the specimen’s instability and failure.

**Fig 8:**
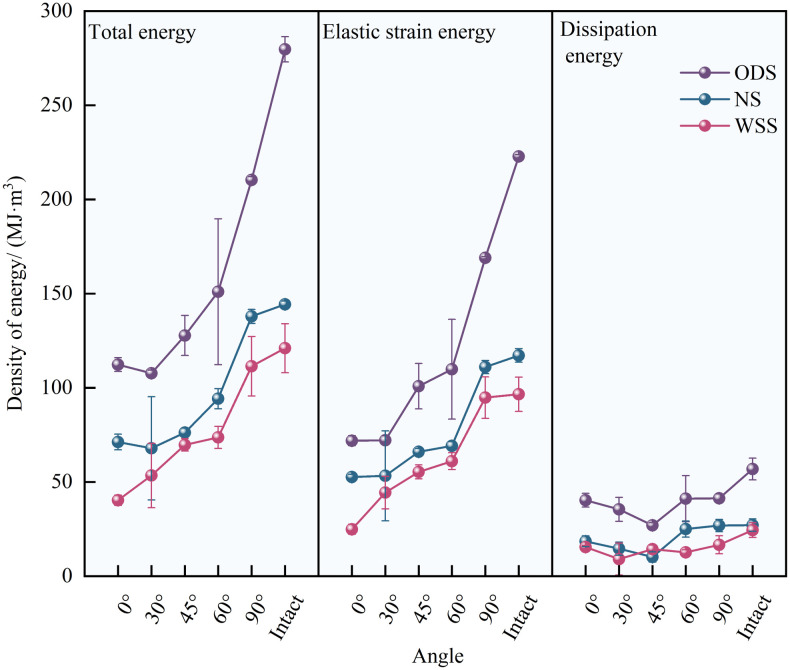
Energy conversion characteristics of fissure rock specimens at the stress peak point under different water conditions.

As shown in Fig 8, the energy evolution characteristics of fissure sandstone specimens at the peak stress point were influenced by the water condition. The total strain energy and elastic strain energy were inversely related to the water content. The existence of water modified the accumulation paths and proportions of total, elastic, and dissipated strain energies, suggesting that water played an inhibitory and regulatory role in the energy evolution process. Notably, the dissipated strain energy of fissure sandstone specimens under oven-dried states was consistently higher than that under natural and water saturated states, while the dissipated strain energy evolution behaviors under the latter two states showed no distinct regular differences. The preceding analysis demonstrated that the energy dissipation behavior of fissure sandstone specimens was strongly dependent on their water state. Because water weakened the mechanical properties of the rock, specimens with lower water content possessed higher internal cohesion, which effectively restrained crack propagation and coalescence, thereby allowing more elastic strain energy to be stored within the rock.

Under oven-dried and natural states, the total strain energy of fissure sandstone specimens exhibited a decreasing–increasing trend with increasing fissure angle, and the minimum value occurred at 30°. However, under water saturated states, the total strain energy showed a distinct increasing tendency with the rise of fissure angle. When the water condition was the same, the elastic strain energy increased noticeably as the fissure angle increased, suggesting that the instability failure of fissure sandstone under energy drive became more difficult with a higher fissure angle. The dissipated strain energy of fissure sandstone specimens varied markedly with fissure angle under different water conditions. In particular, the dissipated strain energy reached the lowest value at a fissure angle of 45° in the oven-dried and natural states, while in the water saturated states, the lowest value occurred at 30°. This analysis demonstrated that the fissure angle modified the accumulation process and distribution pattern of total strain energy, elastic strain energy, and dissipated strain energy, revealing its significant influence on the energy evolution behavior of sandstone specimens.

#### 3.4.2. Energy transformation at crack initiation and damage stresses.

**Fig 9:**
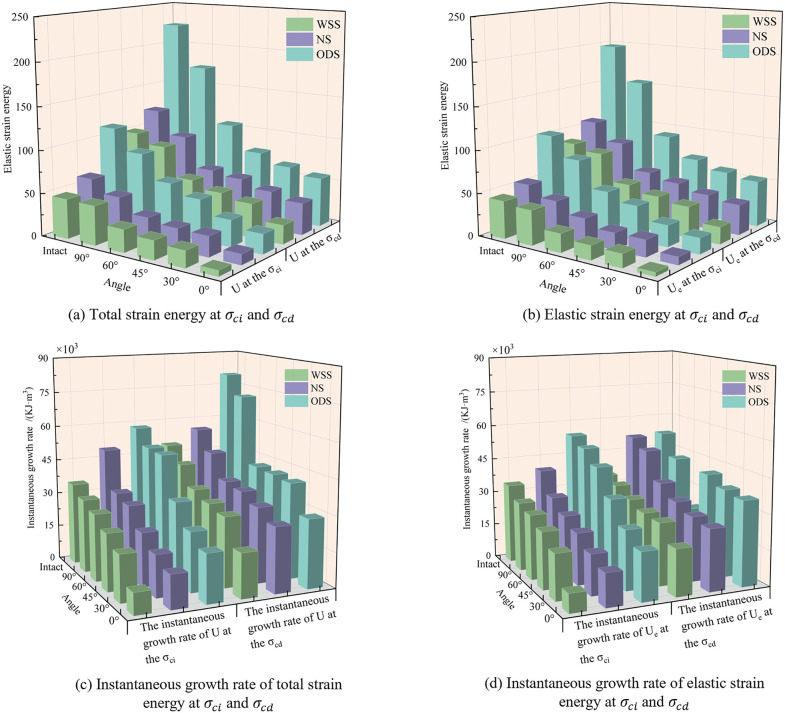
Energy conversion characteristics at crack initiation stress and crack damage stress.

To further analyze the influence of fissure angle and water content on the energy evolution of sandstone, Fig 9 (a) and Fig 9 (b) illustrate the energy evolution patterns at crack initiation stress and crack damage stress. It was observed that, under the same water content, the total strain energy and elastic strain energy exhibited a nonlinear increasing trend with the fissure angle at both crack initiation and damage stresses. The presence of water reduced the total strain energy and elastic strain energy at these stresses. To reflect the activity of energy accumulation and the conversion rate at crack initiation stress and crack damage stress, the instantaneous growth rates of total strain energy and elastic strain energy at these stresses were calculated, as shown in Fig 9(c) and Fig 9(d). The instantaneous growth rates of total strain energy and elastic strain energy at crack initiation stress and crack damage stress increased progressively with fissure angle, exhibiting an overall trend of 0° < 30° < 45° < 60° < 90°. At a fissure angle of 0°, both energy parameters showed the lowest instantaneous growth rates because the stress acted mainly normal to the fissure surface, producing strong compressive closure of the fissure. Under this condition, external energy could not effectively accumulate at the fissure tips. As the fissure angle increased to 30°, 45°, and 60°, the shear stress component along the fissure surface gradually became dominant, leading to continuous increases in the instantaneous growth rates and improving the energy storage efficiency of the rock mass. This behavior indicates a transition in the energy response mechanism from compression-controlled behavior to shear-controlled behavior. At a fissure angle of 90°, the instantaneous growth rates reached their peak values, suggesting the strongest coupling effect between fissure orientation and principal stress direction, as well as pronounced stress concentration. The above results indicate that fissure angle significantly affects the energy evolution characteristics of rocks and enhances the energy storage capacity before macroscopic failure.

It was further observed that, in the majority of cases, the instantaneous growth rate declined progressively as water content increased. In the oven-dried states, the lack of pore water strengthened particle bonding and friction, resulting in energy being mainly stored as elastic strain energy. However, in natural and water-saturated states, water acted to lubricate and soften the rock, reducing particle cohesion and frictional resistance, which led to earlier microcrack slippage and easier energy dissipation at crack tips, thereby markedly decreasing the energy accumulation rate in the rock mass.

#### 3.4.3. Characteristics indicative of imminent failure.

As the rock specimen was loaded, the elastic strain energy stored within it reached its maximum storage capacity, triggering energy release that induced unstable failure. The accumulation of dissipated strain energy intensified internal damage, progressively weakening the rock’s load-bearing ability until its strength was lost. Consequently, rock failure was governed by the coupled action of elastic strain energy and dissipated strain energy, with their ratio (K_ED_) showing a significant dynamic evolution during the entire loading process [39], as described by the following equation.


$$\begin{array}{c} K_{ED}=\frac{U_{e}}{U_{d}} \end{array}$$ (3)

**Fig 10:**
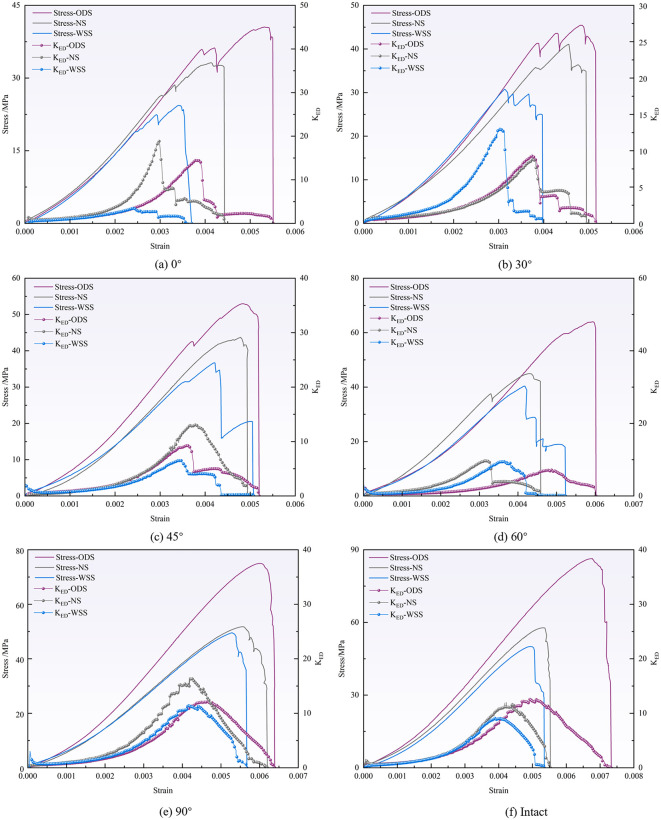
K_ED_ curves of fissure rock specimens under different water content conditions.

K_ED_ represented the ratio of reversible elastic strain energy to irreversible dissipated strain energy within the rock specimen during the loading process, directly reflecting whether the dominant mode of energy conversion involved more energy being stored or irreversibly consumed. Fig 10 illustrates the temporal variation of K_ED_ for fissure sandstone specimens under different water content conditions throughout the entire loading process. It was observed that the K_ED_ curves exhibited distinct peak points during loading, after which dissipated strain energy accelerated due to irreversible damage such as fissure development, causing K_ED_ to decline. The peak of the K_ED_ curve indicated that the dominant mode of energy conversion shifted from “elastic storage-dominated” to “irreversible dissipation-dominated” and occurred before the stress peak, suggesting it was a precursor to rock failure. Therefore, in this study, K_ED_ curves were employed to identify precursor information of rock failure during the yielding stage, thereby enhancing the predictive capability of rock rupture.

Table 3 summarizes the stress peak strain and K_ED_ peak strain of fissured sandstone specimens under different water conditions. In this study, the ratio of K_ED_ peak strain to stress peak strain was defined as a “warning coefficient” for identifying failure precursor information from the K_ED_ curve, thereby reducing the influence of loading conditions on warning results. The expression is given as follows:

**Table 3:** Peak strain and K_ED_ peak strain for fissure rock specimens under different water conditions.

Water condition	Fissure angle	Stress peak strain	K_ED_ peak strain	λ
Oven-dried states	0°	0.00531	0.00377	0.7100
	30°	0.00481	0.00374	0.7775
	45°	0.00482	0.00359	0.7448
	60°	0.00469	0.00375	0.7996
	90°	0.00599	0.00465	0.7763
	Intact	0.00678	0.00510	0.7522
Natural states	0°	0.00412	0.00291	0.7063
	30°	0.00455	0.00377	0.8286
	45°	0.00478	0.00376	0.7866
	60°	0.00255	0.00197	0.7725
	90°	0.00532	0.00423	0.7951
	Intact	0.00557	0.0042	0.7540
Water-saturated states	0°	0.00341	0.00239	0.7009
	30°	0.00313	0.00303	0.9681
	45°	0.00419	0.00341	0.8138
	60°	0.00418	0.00373	0.8923
	90°	0.00529	0.00439	0.8299
	Intact	0.00494	0.00389	0.7874


$$\begin{array}{c} \lambda =\frac{\varepsilon_{\left(K_{ED},max\right)}}{\varepsilon_{\left(\sigma ,max\right)}} \end{array}$$ (4)

Where λ represents the warning coefficient, $\varepsilon_{K_{ED,max}}$ denotes the strain corresponding to the K_ED_ peak, and $\varepsilon_{\left(\sigma ,max\right)}$ denotes the strain corresponding to the stress peak. The warning coefficient characterizes the leading relationship between the K_ED_ peak strain and the stress peak strain and reflects the relative strain warning interval between these two characteristic points. A smaller λ value indicates that the K_ED_ peak appears earlier and that the rock retains a larger strain evolution space before reaching peak failure. Conversely, when λ approaches 1, the K_ED_ peak occurs closer to macroscopic failure, suggesting weaker precursor information and highlighting its potential application in rock failure prediction.

The results in Table 3 indicate that, under the same fissure angle, the warning coefficient generally increased with water content, and λ became closer to 1 under water-saturated states. This finding suggests that increasing water content narrowed the difference between the K_ED_ peak strain and stress peak strain and weakened the precursor identification capability of K_ED_. Moreover, under identical water conditions, the warning coefficient exhibited a typical “M”-shaped variation with fissure angle, with obvious increases at 30° and 60°. Under water saturated states, the specimen with a 30° fissure angle showed the highest λ value of 0.968. This behavior can be attributed to the combined effects of water saturation and a 30° fissure angle, which reduced the effective normal stress on fissure surfaces and enhanced lubrication effects, thereby promoting unstable crack propagation. Under this condition, the specimen also exhibited the minimum strain at peak stress (Fig 3), indicating insufficient crack development during loading and delayed energy dissipation until the failure stage. Consequently, the K_ED_ peak appeared relatively late, and macroscopic failure occurred almost simultaneously with energy mutation. As a result, the precursor information before failure became less evident, reflecting the sudden failure characteristics of the rock. Compared with intact sandstone specimens, fissured sandstone specimens generally showed larger λ values under all fissure angles, indicating that fissures accelerated the failure process and reduced the predictability of rock failure. Furthermore, the strain-based warning coefficient proposed in this study exhibits low sensitivity to loading rate and loading path variations and can be obtained through monitoring methods such as displacement gauges and strain gauges. By integrating long-term monitoring data with empirical prediction models, a real-time strain-based warning system may be established for practical engineering applications.

### 3.5. Failure characteristic analysis

**Fig 11:**
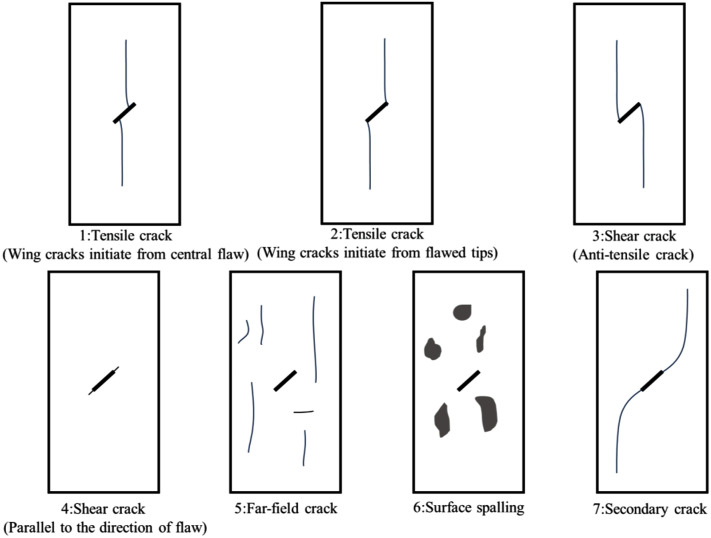
Seven basic types of cracks [35].

Yang et al. [40] classified the cracks generated during rock failure and proposed seven basic types. In contrast to the findings of Yang et al. [40], Zhou et al.[35] categorized the reverse wing cracks (the third type of crack) as shear cracks, and the type II tensile cracks (the seventh type of crack) as secondary cracks, as shown in Fig 11.

Water conditions and fissure angles were key factors that controlled the damage behavior of fissure sandstone. Specifically, water molecules softened the cementation within the rock and reduced interparticle cohesion through physicochemical effects, while fissures governed stress redistribution and crack propagation paths. Their interaction jointly drove the failure characteristics.

**Fig 12:**
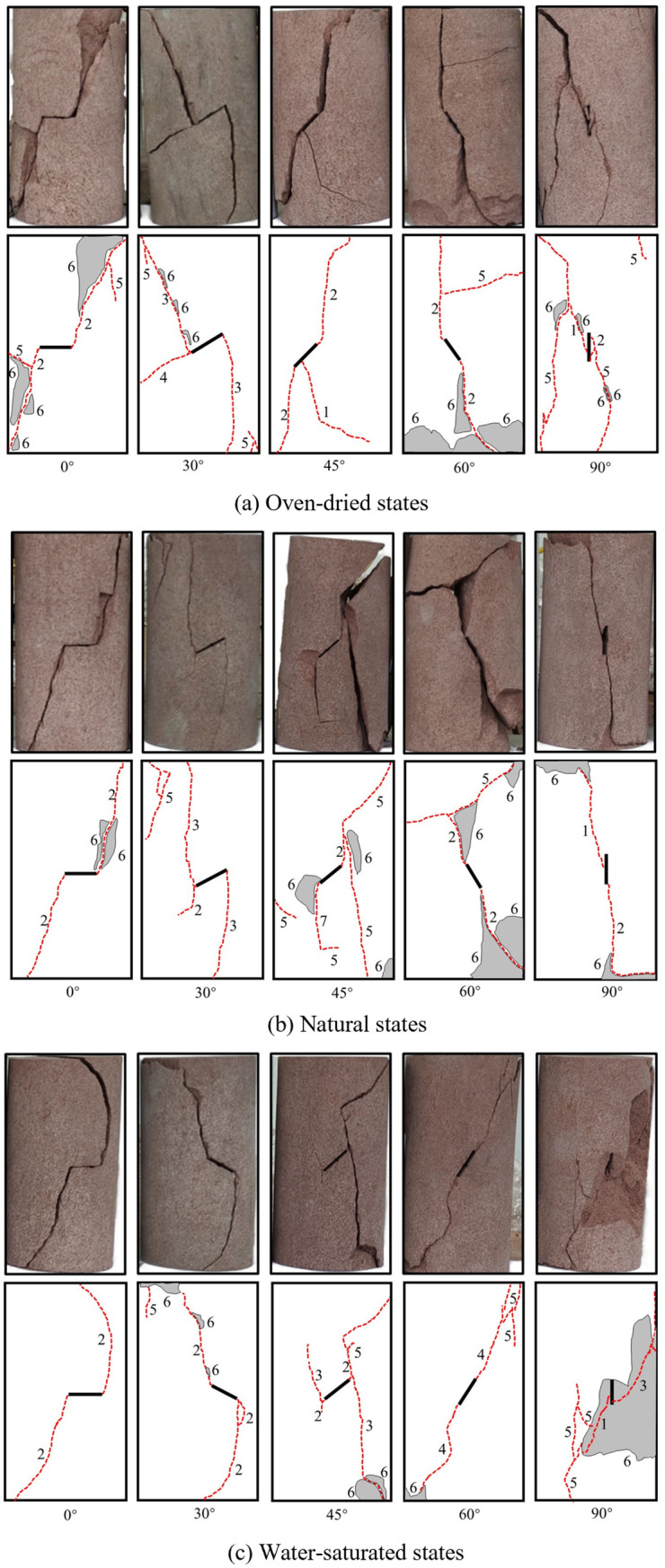
Final failure modes of fissure sandstone specimens under different water contents.

Fig 12 shows the final failure patterns of fissure sandstone specimens under different water conditions. As water content increases, the failure mode of specimens with a fissure angle of 0° changes from complex mixed cracks under dry conditions, including tensile cracks, far-field cracks, and surface spalling, to type II tensile cracks (wing cracks) under saturated conditions. For specimens with a fissure angle of 30°, the failure mode shifts from type III shear cracks (anti-wing cracks) in the dry state to type II tensile cracks under saturated conditions. For fissure angles of 45°and 60°, the failure mode changes from type II tensile cracks to shear cracks. For specimens with a fissure angle of 90°, the failure pattern evolves from tensile–far-field mixed cracks to tensile–shear mixed cracks, both accompanied by surface spalling. Under saturated conditions, the failure mode gradually transitions from tensile-dominated to shear-dominated as the fissure angle increases, because fissures alter stress decomposition and redistribution within the rock, leading to different failure characteristics. Overall, under dry and natural conditions, only the specimen with a fissure angle of 30° exhibited shear-dominated failure, while the others showed type II tensile failure. Under saturated conditions, the specimens with fissure angles of 0°and 30°were dominated by tensile failure, whereas the rest failed in shear. This is primarily because, under dry and natural conditions, crack propagation must overcome tensile stress concentration at the fissure tips. The resulting tortuous crack paths delay energy release, allowing the specimens to accumulate higher elastic strain energy and exhibit higher peak strength. In contrast, under saturated conditions, pore water pressure and the lubricating effect of water molecules reduce the effective normal stress on fissure surfaces and enhance sliding. This shifts crack propagation from tensile-dominated to shear-dominated, leading to a significant reduction in peak strength.

This transition in failure mode further influenced the evolution of characteristic stress. When tensile failure dominated, crack initiation and propagation required overcoming local tensile stress concentration, resulting in higher crack initiation stress and crack damage stress. In contrast, under shear-dominated conditions, crack growth was controlled by fissure sliding, leading to earlier crack development and lower characteristic stress values. Energy evolution also showed distinct patterns. In tensile-dominated failure, elastic strain energy accumulated efficiently during loading, while dissipated strain energy emerged at a later stage, reflecting a typical energy-storage mechanism. The transition from energy storage to irreversible dissipation occurred relatively late, and after this transition, a relatively long crack propagation process followed. As a result, the K_ED_ peak appeared much earlier than the stress peak, providing a larger warning coefficient. In contrast, shear-dominated failure exhibited a dissipation-controlled mechanism, where friction along fissure surfaces and particle rearrangement consumed energy, limiting elastic strain energy accumulation. This reflects a typical energy-dissipation mechanism. The transition to dissipation occurred earlier, and the overall failure process was significantly shortened. Consequently, the K_ED_ peak approached the stress peak, which decreased the warning coefficient.

These results indicated that fissure sandstone under dry conditions exhibited higher characteristic stress, stronger energy storage capacity, and larger warning coefficient than under saturated conditions, which was consistent with the experimental observations. The transition from tensile to shear failure reflected a shift in energy evolution from storage-dominated to dissipation-dominated behavior, which ultimately influenced the variation in characteristic stress and K_ED_-based warning capability. Therefore, water conditions and fissure angle regulated the failure mode of rock, altered the paths of energy accumulation and dissipation, and subsequently affected the mechanical properties and failure precursors, forming a typical mechanical-energy-damage coupled evolution mechanism.

Integrated analysis indicated that type I tensile cracks predominantly developed in sandstone specimens with fissure angles of 45° and 90°. Within this angular range, axial loading induced pronounced lateral tensile stress concentration within the fissure center, which facilitated the preferential propagation of wing cracks. Type I and type II tensile cracks were commonly associated with far-field cracks (type 5). When they coalesced or intersected, the stored elastic strain energy was released rapidly and in a concentrated manner. As a result, the local energy release rate exceeded the dissipation capacity of the rock, inducing surface spalling (type 6). Moreover, surface spalling was also identified in regions where shear cracks (type 3) were highly developed, demonstrating that shear sliding processes could generate localized energy release and stress concentration. These findings confirmed that both tensile- and shear-dominated failure modes led to local instability and surface fragmentation and spalling when energy release became unbalanced or local stress exceeded the load-bearing capacity of the rock.

Based on the above coupling analysis of failure modes, energy evolution, and mechanical properties of fissure sandstone, we established a semi-quantitative criterion for crack propagation modes. The results showed that under dry and natural conditions, specimens with a fissure angle of 30° were dominated by shear failure, whereas other fissure angles mainly exhibited tensile-dominated failure. In contrast, under saturated conditions, specimens with fissure angles greater than 30° were primarily governed by shear failure. Therefore, a fissure angle of approximately 30° represented a critical interval for the transition between tensile- and shear-dominated failure modes. Within this range, sandstone specimens showed high sensitivity to changes in stress conditions. Further comparison indicated that specimens with a fissure angle of 30° were dominated by shear failure under dry conditions but shifted to tensile-dominated failure under saturated conditions, demonstrating that water molecules significantly altered the failure mode within this critical range. Due to the limited classification of water conditions in this study, we did not determine the critical interval for the transition between tensile and shear failure modes under varying water contents. Future work will refine water content gradients and employ multiscale experimental approaches to systematically investigate the critical interval of water conditions and its coupling relationship with fissure angle. This effort will provide important insights into the coupling damage mechanism between water and fissures.

### 3.6. Interaction between water conditions and fissure angle

To further clarify the coupling effect between water conditions and fissure angle, the influence rates of mechanical parameters under natural states and water saturated states relative to oven-dried states were calculated for different fissure angles. The analyzed parameters included characteristic stress and energy evolution characteristics. The corresponding equations are expressed as follows.

The influence rate of natural states relative to oven-dried states:


$$\begin{array}{c} \eta_{natural}=\frac{X_{dry}-X_{natural}}{X_{dry}}\times 100\% \end{array}$$ (5)

The influence rate of water saturated states relative to oven-dried states:


$$\begin{array}{c} \eta_{saturated}=\frac{X_{dry}-X_{saturated}}{X_{dry}}\times 100\% \end{array}$$ (6)

Where $X_{dry}$, $X_{natural}$, and $X_{saturated}$ denote the experimentally measured values of a given mechanical parameter under oven-dried states, natural states, and water saturated states, respectively. The parameters $X_{natural}$ and $X_{saturated}$ quantify the weakening effect of water on the mechanical properties of fissured sandstone, and larger values indicate more pronounced water-induced deterioration.

**Fig 13:**
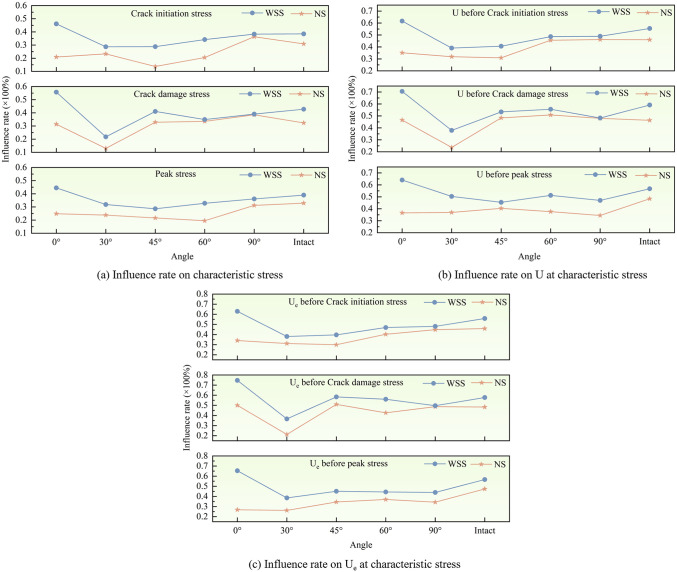
Influence rates of mechanical parameters under natural states and water saturated states relative to oven-dried states at different fissure angles.

Fig 13 shows that $X_{saturated}$ is consistently greater than $X_{natural}$, while their variation trends with fissure angle differ significantly. Moreover, the difference between them exhibits a non-monotonic evolution pattern with fissure angle. These results indicate that the weakening effect of water on fissured sandstone increases with water content and is strongly influenced by fissure angle. To better characterize the coupling effect between water conditions and fissure angle, this section selected oven-dried states and water-saturated states as two extreme boundary conditions and focused on the influence rates of mechanical parameters under water-saturated states relative to oven-dried states. The results in Fig 13(a) demonstrate that the influence rate of water on peak strength reached 44.51% at a fissure angle of 0°, whereas the minimum value of 28.59% occurred at 45°, showing a clear non-monotonic trend. At a fissure angle of 30°, the influence rates of water on crack initiation stress and crack damage stress under water saturated states were 21.78% and 28.68%, respectively, which were lower than those under other fissure angles. This finding suggests that specimens with a 30° fissure angle exhibited relatively low sensitivity to water. Under this condition, crack initiation and propagation were still dominated by mechanical effects, and the softening effect of water remained relatively weak.

Furthermore, the weakening effect of water on the total strain energy and elastic strain energy of fissure sandstone is also closely related to fissure angle, as illustrated in Fig 13(b) and Fig 13(c). At a fissure angle of 30°, the influence rate of water on the total strain energy corresponding to the crack initiation stress is 37.86% under water saturated states, which is lower than that observed at other fissure angles. The elastic strain energy at the crack damage stress shows the same tendency. This finding suggests that the 30° specimen possesses relatively low water sensitivity, and the energy accumulation required for crack initiation and propagation remains comparatively stable even in the presence of water. By comparison, the specimen with a 0° fissure angle is highly sensitive to water. Under water saturated states, the influence rate of water on the total strain energy at the crack initiation stress reaches as high as 70.50%. Similar trends are also identified in the elastic strain energy at the crack damage stress and in both the total strain energy and elastic strain energy at the peak stress. These results confirm that water markedly reduces the energy threshold required for crack initiation and propagation when the fissure angle is 0°.

This coupling behavior is closely related to the stress condition at the fissure tip. At a fissure angle of 0°, axial stress acts normal to the fissure plane, causing the fissure surface to mainly sustain compressive stress. As a result, significant tensile stress concentration develops easily at the fissure tip. The combined action of water lubrication and tensile stress concentration facilitates crack initiation and propagation, thereby substantially degrading the mechanical properties and energy storage capacity of the sandstone specimen. Accordingly, the 0° specimen exhibits the strongest water sensitivity, which is manifested by the highest influence rates of water on all mechanical and energy parameters under water-saturated states. At a fissure angle of 30°, however, the shear stress component at the fissure tip increases significantly, and fissure sliding becomes more active. Although water lubrication reduces frictional resistance, fissure sliding accelerates energy release and stress redistribution, thereby weakening the softening effect induced by water. Consequently, the 30° specimen exhibits relatively weak sensitivity to water. For the specimens with fissure angles of 45°, 60°, and 90°, crack propagation paths become more complicated, and the interaction between water and various mechanical mechanisms produces no distinct regular pattern in the overall weakening effect. Therefore, water conditions and fissure angle do not independently affect the mechanical behavior of fissured sandstone. Instead, they jointly regulate the stress state at the fissure tip and the energy evolution process, resulting in a pronounced coupling effect.

## 4. Discussion

Based on the experimental data, a statistical analysis of the warning coefficient was performed to establish a preliminary risk classification criterion. The statistical results presented in Table 3 show that the mean value of λ is μ = 0.793 and the standard deviation is β = 0.064. Approximately 72% of the data are distributed within the interval [μ − β, μ + β] = [0.73, 0.86], while approximately 94% fall within [μ − 2β, μ + 2β] = [0.666, 0.924]. The overall distribution pattern is generally consistent with the empirical characteristics of a normal distribution, corresponding approximately to the 68% and 95% statistical intervals. Accordingly, the risk classification intervals of the warning coefficient were established according to the statistical distribution characteristics, thereby providing a statistical basis for the proposed risk classification method. Accordingly, the warning coefficient was divided into three intervals: a low-risk interval (λ < 0.73), a medium-risk interval (0.73 ≤λ  < 0.86), and a high-risk interval (0.86 ≤λ  ≤ 1). It should be emphasized that the proposed classification criterion mainly reflects the relative variation in warning capability and instability abruptness under different experimental conditions, rather than representing an absolute criterion for rock instability failure.

Based on the above classification criterion, approximately 16% of the specimens are classified into the low-risk interval, approximately 73% belong to the medium-risk interval, and approximately 11% fall into the high-risk interval. When the warning coefficient remains within the low-risk interval, the sandstone specimen still possesses a relatively large strain evolution space before reaching peak stress, and the precursory characteristics prior to failure are relatively distinct. Once the warning coefficient enters the medium-risk interval, the sandstone specimen reaches a critical stage of damage evolution, indicating that monitoring frequency should be strengthened. When the warning coefficient further approaches the high-risk interval, the precursory information before failure becomes considerably weakened, and therefore, real-time monitoring should be further enhanced. Furthermore, the warning coefficient gradually shifts toward the high-risk interval with increasing water content, indicating that water weakens the capability of the K_ED_ peak to identify precursory information associated with rock failure. It should also be noted that the above threshold values were preliminarily established under the specific experimental conditions of this study, including lithology and fissure geometry. Therefore, they should be regarded as a reference framework rather than a universal criterion. In practical geotechnical engineering applications, the strain-based warning coefficient can be determined through real-time deformation monitoring methods, such as displacement meters and strain gauges. Combined with field monitoring data, historical instability cases, and geological conditions, the warning thresholds can be dynamically calibrated, and the risk classification can be further optimized.

Although the present study mainly investigated the qualitative evolution characteristics of the mechanical-energy-damage coupling mechanism, the experimental results provide an important foundation for developing a quantitative instability prediction framework for fissured sandstone under the interaction between water and fissures. Based on the obtained results, a preliminary quantitative coupling framework was further proposed to characterize the instability evolution of fissured sandstone through the coupled response of mechanical parameters, energy evolution characteristics, and damage variables. Specifically, the fissure angle (α) primarily governs stress redistribution and crack propagation paths within sandstone specimens, whereas the water condition parameter (ω) affects effective normal stress and inter-particle bonding strength through lubrication effects and physicochemical softening mechanisms. The interaction of these factors consequently controls the evolution of elastic strain energy accumulation and dissipated energy release. Therefore, the following preliminary quantitative framework was introduced:


$$\begin{array}{c} D=f(\alpha ,\omega ,U_{d}/U) \end{array}$$ (7)

Where D denotes the damage variable, α represents fissure angle, ω denotes the water condition parameter, and $U_{d}/U$ corresponds to the ratio of dissipated strain energy to total strain energy. The proposed framework reflects the coupled effects of fissure geometry, water-induced weakening, and energy dissipation evolution on the instability behavior of fissured sandstone. Furthermore, by incorporating the warning coefficient λ proposed in this study, the framework provides a preliminary quantitative basis for instability prediction of fissured sandstone. Nevertheless, the proposed model framework still requires systematic validation and parameter calibration using a large amount of experimental data obtained from different lithologies, water conditions, and fissure geometries. Future studies will combine acoustic emission monitoring, mesoscopic numerical simulations, and multi-field coupling analysis to establish a more comprehensive and unified quantitative prediction model for fissure sandstone instability and failure.

Further investigation into the crack propagation mechanism of fissured sandstone under the interaction between water and fissures can enhance the understanding of coupled multi-physical failure mechanisms in rock masses and provide meaningful guidance for improving the operational safety and long-term stability of geotechnical engineering. The study results reveal that increasing water content gradually weakens the characteristic stress and energy storage capability of fissured sandstone specimens, and the overall failure mode progressively changes from tensile-dominated failure to shear-dominated failure. Under low water conditions, wing cracks mainly govern the internal crack propagation process. Therefore, surface protection and spraying measures can effectively delay crack initiation and crack growth. In water-rich engineering regions such as tunnels, slopes, and foundation excavations, greater attention should be paid to mitigating the lubrication and softening effects of water on fissure surfaces. Measures including grouting treatment, drainage, depressurization, and seepage prevention control can effectively reduce water-induced deterioration. In addition, rock bolts can improve the shear resistance of fissure surfaces and strengthen the integrity of the rock mass. Moreover, the K_ED_ peak approaches the stress peak under some fissure angle conditions, indicating stronger sudden failure characteristics and a shorter warning period before instability. Accordingly, λ can be determined in real time through monitoring methods such as displacement sensors and strain gauges in practical engineering applications. When λ remains within the low-risk interval, the rock mass still exhibits obvious precursory failure information, and standard monitoring measures can be maintained. After λ enters the medium-risk interval, monitoring frequency and field inspections should be increased. When λ reaches the high-risk interval, engineers should promptly issue warning signals and further strengthen field monitoring and stability assessment. In this study, the warning coefficient λ of several specimens under water saturated states reached 0.968, reflecting significant sudden failure characteristics. Therefore, rock masses near certain fissure angles in water-rich engineering areas should receive special attention during field investigation and monitoring. Overall, these targeted prevention and control strategies can provide practical guidance for disaster mitigation and risk assessment in fissured rock engineering under the interaction between water and fissures.

It is important to note that the present study only employed uniaxial compression tests, while actual rock masses in geotechnical engineering commonly experience complicated confining pressure environments. Therefore, future research will conduct conventional triaxial compression tests under different confining pressure conditions to further clarify the mechanical response, energy evolution, and failure behavior of fissure sandstone subjected to the interaction between water and fissures under confinement. In this study, only three water conditions, including oven-dried states, natural states, and water saturated states, were considered. Consequently, the critical water content threshold corresponding to characteristic stress degradation and the transformation from tensile failure to shear failure has not yet been established. Future investigations will introduce continuous water content gradients to accurately determine the critical threshold value. In addition, the indicators $R_{ci}$ and $R_{cd}$ proposed in this paper were not designed to replace traditional damage variables. Instead, they were introduced as supplementary parameters to quantify the weakening effects of fissures on crack initiation stress and crack damage stress. Moreover, the experiments in this study only focused on one type of sandstone. Thus, the applicability of indicators $R_{ci}$, $R_{cd\;}$, and their corresponding evolution characteristics under different lithological conditions still requires further validation. Comparative studies involving granite, limestone, and other rock types will therefore be conducted in future work. Meanwhile, the current study included a relatively limited number of experimental groups. Future research will increase the sample size and adopt stricter statistical analyses to enhance the reliability and robustness of the experimental findings. Finally, the present study mainly evaluated fissured sandstone based on characteristic stress, energy evolution, and macroscopic failure patterns. In future work, acoustic emission monitoring, CT scanning, and numerical simulation techniques will be integrated to investigate crack evolution and energy dissipation mechanisms under the interaction between water and fissures from a multi-scale perspective, thereby providing deeper insight into the intrinsic physical mechanisms underlying the macroscopic mechanical behavior.

## 5. Conclusion

This study employed uniaxial compression tests to investigate the effects of water content and fissure angle on the mechanical properties, energy evolution, and failure patterns of sandstone. The following main conclusions were drawn from this work.

(1) The variations in water content and fissure angle induced pronounced anisotropy in the crack initiation stress, crack damage stress, and peak strength of the sandstone samples. The relationship between peak strength and fissure angle can be fitted using a nonlinear function, while peak strain exhibited a “W”-shaped variation as the fissure angle increased. In the oven-dried states, fissures reduced the peak strength of the sandstone more than in the water-saturated states, and when the fissure orientation deviated from the loading direction by up to 30° (α ≤ 30°), the reduction in peak strength reached 30%.
(2) A new parameter for characteristic stress weakening was applied to improve the evaluation of rock damage. The findings indicated that the degree of weakening of characteristic stress decreased with increasing fissure angle, and smaller fissure angles exerted more significant weakening effects. Across varying water content conditions, fissures affected the crack initiation phase more than the stable crack propagation phase.
(3) Water content and fissure angle suppressed the total strain energy and elastic strain energy at characteristic stress, and altered their accumulation paths and distribution ratios. The instantaneous growth rates of total and elastic strain energy at the crack initiation stress and crack damage stress were positively correlated with fissure angle, while the presence of water reduced these growth rates. Increasing water content markedly narrows the gap between the K_ED_ peak strain and the stress peak strain, which consequently weakens the effectiveness of K_ED_ in identifying failure precursor characteristics. Meanwhile, the warning coefficient of fissured sandstone specimens shows a characteristic “M”-shaped variation pattern as the fissure angle increases.
(4) Fissures altered the redistribution of internal stresses in sandstone, and variations in fissure angle affected its failure characteristics. The softening effect of water modified the adhesion and friction between particles, influencing the failure modes of fissure sandstone. Therefore, the coupling of water and fissures jointly affected the damage evolution and failure characteristics of the rock.

## Supporting information

S1 File Minimum data set. (ZIP)
